# Field study of the improved rapid sand fly exposure test in areas endemic for canine leishmaniasis

**DOI:** 10.1371/journal.pntd.0007832

**Published:** 2019-11-21

**Authors:** Laura Willen, Tereza Lestinova, Barbora Kalousková, Petra Sumova, Tatiana Spitzova, Rita Velez, Ester Domenech, Ondřej Vaněk, Montserrat Gállego, Pascal Mertens, Petr Volf

**Affiliations:** 1 Department of Parasitology, Faculty of Science, Charles University, Prague, Czech Republic; 2 Department of Biochemistry, Faculty of Science, Charles University, Prague, Czech Republic; 3 ISGlobal, Hospital Clínic—Universitat de Barcelona, Barcelona, Spain; 4 Secció de Parasitologia, Departament de Biologia, Sanitat i Medi Ambient, Facultat de Farmàcia i Ciències de l’Alimentació, Universitat de Barcelona, Barcelona, Spain; 5 Hospital Veterinari Canis, Girona, Spain; 6 Coris BioConcept, Crealys Park, Gembloux, Belgium; Universidade Federal de Minas Gerais, BRAZIL

## Abstract

**Background:**

Canine leishmaniasis (CanL) is a severe chronic disease caused by *Leishmania infantum* and transmitted by sand flies of which the main vector in the Western part of the Mediterranean basin is *Phlebotomus perniciosus*. Previously, an immunochromatographic test (ICT) was proposed to allow rapid evaluation of dog exposure to *P*. *perniciosus*. In the present study, we optimized the prototype and evaluated the detection accuracy of the ICT in field conditions. Possible cross-reactions with other hematophagous arthropods were also assessed.

**Methodology/Principal findings:**

The ICT was optimized by expressing the rSP03B protein in a HEK293 cell line, which delivered an increased specificity (94.92%). The ICT showed an excellent reproducibility and inter-person reliability, and was optimized for use with whole canine blood which rendered an excellent degree of agreement with the use of serum. Field detectability of the ICT was assessed by screening 186 dogs from different CanL endemic areas with both the SGH-ELISA and the ICT, and 154 longitudinally sampled dogs only with the ICT. The ICT results corresponded to the SGH-ELISA for most areas, depending on the statistical measure used. Furthermore, the ICT was able to show a clear seasonal fluctuation in the proportion of bitten dogs. Finally, we excluded cross-reactions between non-vector species and confirmed favorable cross-reactions with other *L*. *infantum* vectors belonging to the subgenus *Larroussius*.

**Conclusions/Significance:**

We have successfully optimized the ICT, now also suitable to be used with whole canine blood. The test is able to reflect the seasonal fluctuation in dog exposure and showed a good detectability in a field population of naturally exposed dogs, particularly in areas with a high seroprevalence of bitten dogs. Furthermore, our study showed the existence of favorable cross-reactions with other sand fly vectors thereby expanding its use in the field.

## Author summary

Canine leishmaniasis (CanL) is a widespread severe chronic disease in dogs, caused by the protozoan parasite *Leishmania infantum*. *Leishmania* parasites are transmitted by the bite of phlebotomine sand flies, of which the most important one in southwestern Europe is *Phlebotomus perniciosus*. A sand fly bite is accompanied by the inoculation of saliva, which elicits a specific antibody response in the host. Past studies have used these antibodies as markers of exposure to sand flies. Recently, a rapid sand fly exposure test was prepared using one salivary protein of *P*. *perniciosus*, the SP03B protein. This rapid test possessed a high sensitivity and specificity when screening experimentally exposed versus non-bitten dogs. However, since naturally exposed dogs possess a higher variability of antibodies against sand fly saliva, we have optimized the previously proposed rapid test and evaluated its detectability on field samples. In this study we present the optimized rSP03B sero-strip that possesses a higher specificity and an ability to detect seropositive dogs from a naturally exposed dog population in a similar fashion as standard serological methods. Furthermore, we have optimized it with whole canine blood which renders it suitable for use in field conditions.

## Introduction

*Leishmania infantum* is a protozoan kinetoplastid parasite (Trypanosomatida, Trypanosomatidae) that annually accounts for approximately 1200 to 2000 new cases of human visceral leishmaniasis (VL) in the Mediterranean basin [1]. It is a zoonotic disease transmitted by sand flies (Diptera, Psychodidae), with the most important vector in the western part of the Mediterranean basin being *Phlebotomus perniciosus* [2]. Domestic dogs represent the primary reservoir; most infected dogs will appear asymptomatic, others may suffer from a severe chronic disease known as canine leishmaniasis (CanL) [3]. Importantly, both clinical and subclinical infections contribute to the transmission of CanL [3,4]. Across the Mediterranean, canine seroprevalences of the infection vary greatly according to region, going from 3.7–34.6% in Spain [5] and from 3.9–24% in Italy [6–8]. However, these numbers underestimate the real amount of infected dogs in the area as has been confirmed by PCR-based screening of seronegative dogs [9,10]. Importantly, municipal kennels housing dogs throughout their life are frequently found in certain European countries and contribute to the persistence of focal hot spots of CanL, thereby forming a major barrier to the control of the disease [11].

The zoonotic nature of the disease together with the severe clinical symptoms it may cause in dogs calls for efforts to control the vector and the spread of the infection. Hence, information on sand fly population dynamics is fundamental and can be obtained by measuring vector exposure in hosts living in endemic areas. This has already been achieved in numerous previous studies by using whole sand fly salivary gland homogenate (SGH) [12,13] or specific salivary proteins [14–17]. The IgG response against the *P*. *perniciosus* salivary protein SP03B has been shown to relate to the seasonal abundance of this sand fly species [14] and to be highly reactive in dogs bitten by different *P*. *perniciosus* populations across countries in the Mediterranean area [18,19]. All these studies together verify the use of the IgG response against this protein as a marker of exposure, capable to replace the use of SGH.

In order to allow rapid evaluation of vector control programs, our recent study focused on using the specific IgG response against the *P*. *perniciosus* SP03B salivary protein as the detectable agent in a rapid vector exposure test, the rSP03B sero-strip [20]. The serological immunochromatographic test (ICT) was prepared with the recombinant SP03B protein purified from overexpression in *E*. *coli* and showed to be a valid replacement for the standard ELISA method. Even though the predictive values of this ICT were already in the excellent range–with a sensitivity of 100% and a specificity of 86.8%–further efforts to increase the specificity of the test were undertaken. In the present study we have optimized the ICT by using the rSP03B recombinant salivary protein expressed in the Human Embryonic Kidney (HEK) 293S GnTI^-^ cell line. The use of this cell line was based on work previously performed on the yellow-related salivary proteins of *Lutzomyia longipalpis* [16,17]. Furthermore, in order to facilitate the use of the rSP03B sero-strip in the field, it was optimized for use with whole canine blood. Moreover, we assessed the detection accuracy of the rSP03B sero-strip in field conditions by screening canine populations exposed to various frequencies of sand fly bites in different areas in the Mediterranean basin and collected on different time points. In order to exclude unfavorable cross-reactions between the proposed rSP03B sero-strip and non-vector sand fly species together with other hematophagous arthropods present in the Mediterranean basin, we tested the cross-reactivity between salivary antigens of *P*. *perniciosus* (whole SGH and rSP03B protein) and sera of animals that were experimentally bitten by *Ixodes ricinus*, *Ctenocephalides felis*, *Culex molestus*, *P*. *papatasi*, *P*. *sergenti* or *P*. *tobbi*.

## Methods

### Ethics statement

Ethical approval for the reuse of canine sera samples from previous studies [13,14,18] was granted by the Ethical Board of Charles University (Prague, Czech Republic). The use of canine sera samples obtained from non-bitten laboratory-bred Beagles housed at the University of Zaragoza, Spain (UNIZAR) was approved during another ongoing study, protocol PI44/17. Collection of sera samples of naturally exposed field dogs was performed by a trained veterinarian and did not include additional or unnecessary invasive procedures. Consent was obtained from the dog owners. International animal experimentation guidelines were followed. All sampling complied with the European guidelines on the protection of animals (Directive 2010/ 63/UE). BALB/c mice were maintained and handled in the animal facility of Charles University (Prague, Czech Republic) following institutional guidelines and Czech legislation (Act No. 246/1992 and 359/2012 coll. on the Protection of Animals against Cruelty in present statutes at large), complying with all relevant European Union and international guidelines for experimental animals. All experiments were approved by the Committee on the Ethics of Laboratory Experiments of the Charles University (Prague, Czech Republic) (permission no. MSMT-10270/2015-6 of the Ministry of the Environment of the Czech Republic).

### Sources of samples

In order to assess the predictive values of the optimized rSP03B sero-strip, the test was run with 119 sera samples from laboratory-bred Beagles, being 60 sera samples from dogs experimentally exposed to *P*. *perniciosus*, 30 sera samples from non-bitten laboratory-bred Beagles housed in a breeding facility in northern France, and 29 sera samples from non-bitten laboratory-bred Beagles housed at the University of Zaragoza, Spain (UNIZAR). The sampling protocol of canine sera samples obtained from dogs individually exposed to approximately 200 *P*. *perniciosus* females is described in more detail in [13]. Furthermore, the test was optimized for the use of whole canine blood by spiking a blood sample collected from a healthy unexposed dog living in the Czech Republic with sera samples.

Furthermore, to evaluate the field detection capability of the rSP03B sero-strip, sera samples from naturally bitten dogs in regions endemic for CanL were used. More specifically, sera samples from naturally-exposed dogs housed in open-air kennels in rural areas in Montagut and Canet d’Adri (province of Girona, Catalunya, Spain) collected in July (n = 29 and n = 12) and September 2017 (n = 26 and n = 12), respectively were evaluated. Furthermore, canine sera samples from 12 naturally exposed dogs of different breeds living in domestic gardens in Colònia de Sant Jordi (Ses Salines District, Mallorca, Spain) were analyzed. Additionally, 38 sera samples from dogs living in Campania (Italy) collected in July 2012 were tested, as well as a total of 57 canine sera samples collected in Umbria (Italy) at different points in time (October 2013 (n = 27), August 2013 (n = 30)). The sampling protocol of the samples derived from Campania and Umbria is explained in more detail in [18]. For all samples listed above, no pre-selection of dogs took place in order to determine the performance of the test in the field as close as possible. Dogs from both Spain and Italy comprised a mixture of hunting breeds and mongrels. Furthermore, sera samples from 154 naturally exposed dogs housed in an open-air kennel in a rural municipality of Naples province (Campania region, southern Italy) sampled multiple times during two transmission seasons were used [14] to evaluate the previously demonstrated seasonal fluctuation of the anti-*P*. *perniciosus* Ab response with the rSP03B sero-strip.

Finally, to assess potential cross-reactions with other hematophagous arthropods, sera samples from mice or dogs experimentally exposed to *C*. *felis*, *I*. *ricinus*, *Cu*. *molestus*, *P*. *papatasi*, *P*. *sergenti*, *P*. *tobbi*, and *P*. *perniciosus* were used. More specifically, canine sera samples from laboratory-bred Beagles six times experimentally exposed to several hundreds of *C*. *felis* were used to screen for cross-reactions between specific anti-*C*. *felis* IgG and salivary proteins (SGH and rSP03B) from *P*. *perniciosus*. Furthermore, sera samples from mice exposed twice to ten *I*. *ricinus* nymphs were used to test for a reaction with specific anti-*I*. *ricinus* IgG. Lastly, sera samples positive for anti-*Culex* and anti-*Phlebotomus* antibodies were collected from BALB/c mice used for regular blood feeding of mosquito (*Cu*. *molestus*) and sand fly (*P*. *papatasi*, *P*. *sergenti*, *P*. *tobbi*, *P*. *perniciosus*) colonies maintained in the insectary at Charles University (Prague, Czech Republic) according to [21]; all of them being exposed more than ten times. Sera from non-bitten mice were used as negative control.

### Antigens

The antigen used for the preparation of the optimized rSP03B sero-strip was the recombinant 43 kDa yellow-related protein of *P*. *perniciosus* (SP03B, GenBank accn. DQ150622) expressed in a human cell line. Cloning, production and purification of this protein was performed by isolating the mRNA coding for SP03B from one-day-old *P*. *perniciosus* females by the High Pure RNA Tissue Kit (Roche), after which it was transcribed into the cDNA by Transcriptor First Strand cDNA Synthesis Kit (Roche). The DNA fragment was amplified by PCR and subcloned into the pTW5sec expression plasmid, a derivative of pTT5 [22,23]. Proteins expressed from this plasmid contain amino acid residues ITG at their N-terminus and GTHHHHHHHHG, i.e. a histidine tag, at their C-terminus. The rSP03B protein was transiently expressed in HEK293S GnTI^-^ cells (ATCC CRL-3022) as previously described in [23]. Briefly, suspension adapted HEK293S GnTI^-^ cells were grown in EX-CELL293 medium (square-shaped glass bottles) supplemented with 4 mM L-glutamine (Sigma) and shaken at 135 rpm in a humidified incubator at 37°C with 5% CO_2_. For transient transfection, the cell culture was freshly transferred into EX-CELL293 medium at 20 × 10^6^ cells/ml cell density. The expression plasmid DNA (diluted in PBS; 1 μg of DNA per 1 × 10^6^ cells) and the 25 kDa linear polyethylenimine were added directly in a 1:4 weight ratio into the high-density cell culture. Following 4 hours of incubation, the culture was diluted with EX-CELL293 medium to 2 × 10^6^ cells/ml. Culture medium was harvested five to seven days post-transfection by centrifugation (10000 × g, 30 min) and filtered thereafter (0.22 μm Steritop filter; Millipore, USA). Before purification, the harvested medium was diluted with an equal volume of buffer (50 mM Na_2_HPO_4_, 300 mM NaCl, 10 mM NaN_3_, pH 7.5). The histidine-tagged product was then recovered by IMAC chromatography on HiTrap TALON crude columns (GE Healthcare, USA) and further purified by size exclusion chromatography on Superdex 200 10/300 GL column (GE Healthcare, USA). Finally, the protein identity was verified by mass spectrometry. The UV absorbance value of the protein was measured using a nanospectrophotometer at 280 nm. Protein concentration was then quantified by means of its known theoretical molar extinction coefficient.

The whole SGH from *P*. *perniciosus* was used as antigen in ELISA to serve as the gold standard against which the rSP03B sero-strip is evaluated. A colony of *P*. *perniciosus* was reared under standard conditions as described in [21]. Four-to-six day-old female sand flies were dissected; salivary glands were collected and pooled in 20 mM Tris buffer with 150 mM NaCl (TBS). The SGH were stored at -20°C and prepared before use by disrupting the salivary glands during three freeze-and-thaw cycles in liquid nitrogen.

### Indirect enzyme-linked immunosorbent assay

An indirect enzyme-linked immunosorbent assay (ELISA) was performed on 186 sera samples to screen for anti-*P*. *perniciosus* IgG. The ELISA was carried out analogously to a previous study [20]. In short, *P*. *perniciosus* salivary gland homogenate (SGH) was coated on flat bottom microtiter plates (Immulon; 0.2 salivary gland per well) in 20 mM carbonate-bicarbonate buffer (pH 9, 100 μl/well) and incubated overnight at 4°C. After blocking the plates with 6% (w/v) low fat dry milk diluted in PBS + 0.05% Tween 20 (PBS-Tw), canine sera diluted 1/200 in 2% (w/v) low fat dry milk/PBS-Tw were added to the wells (100 μl/well). Further, the plates were incubated with secondary antibodies (polyclonal anti-dog IgG-horseradish peroxidase (HRP), Bethyl laboratories, 100 μl/well) diluted 1:9000 in PBS-Tw. The ELISA was developed with orthophenylendiamine (OPD) in a phosphate-citrate buffer (pH 5.5) and 0.1% hydrogen peroxide. The reaction was stopped after 5 min with 10% sulfuric acid and the absorbance (OD value) was measured at 492 nm using a Tecan Infinite M200 microplate reader (Schoeller). Each serum was tested in duplicate.

### Principle of the rSP03B sero-strip

The principle of the rSP03B sero-strip has been described in our previous study [20]. In essence, the serum sample to be tested is deposited (1 μl) on the sample line of the rSP03B sero-strip immediately after a blocking buffer (3 μl) is applied on the same line. Both the buffer and the sample migrate to the upper part of the strip, where the anti-rSP03B antibodies present in the sample bind to the rSP03B coated on the test line. After dipping the strip into the migration buffer (120 μl), the colloidal gold conjugated to anti-dog IgG (gold-conjugate) starts migrating upwards, eventually leading to labeling of the dog IgG captured on the test line by the immobilized rSP03B. The control colloidal gold conjugate (chicken antibody) will bind to the goat-anti chicken antibodies present at the control line. In case of a positive sample, two purple lines will appear on the nitrocellulose (NC) membrane, whereas for negative samples only the control line will be visible. In the present study the ICT was further optimized for use with whole canine blood to allow faster and immediate screening of samples in the field without the need for serum preparation. In order to do so, a blood sample from a healthy non-exposed dog living in the Czech Republic was spiked (2 units cells: 1 unit serum) with 30 sera samples from experimentally exposed laboratory-bred Beagles and 29 non-bitten laboratory dogs from Zaragoza (Spain) to define the predictive values of the test when whole canine blood is used. The same blood sample was also spiked with sera samples coming from dogs living in an open-air kennel in Montagut and Canet d’Adri (province of Girona, Catalunya, Spain), and dogs living in domestic gardens in Colònia de Sant Jordi (Ses Salines District, Mallorca, Spain) in order to compare the functionality of the test when sera or blood samples are used in CanL endemic settings. For optimal performance of the strip, small adjustments in the lay-out of the strip had to be made; (1) the blood sample (1 μl) is deposited immediately on the sample line without the need of saturating the NC membrane with the blocking buffer, and (2) the anti-dog IgG gold-conjugate was impregnated at a higher concentration compared to when serum is used.

### Cross-reactivity with other hematophagous arthropods

A western blot analysis was performed to study cross-reactions between the salivary proteins of all previously mentioned arthropods and *P*. *perniciosus*. Both *P*. *perniciosus* SGH (15 glands per well; 0.22 ug of protein per gland) and the rSP03B protein (46 μg/ml) were separated on a 12% SDS–PAGE gel under non-reducing conditions using the Mini-Protean III apparatus (BioRad) and blotted on an NC membrane. Subsequently, the NC membrane was cut into strips which were blocked with 5% low fat dry milk in TBS + 0.05% Tween 20 (pH 7.2, TBS-Tw) and incubated for 1 hour with sera diluted at 1:50 in TBS-Tw for sera positive for *I*. *ricinus* and *C*. *felis*; at 1:200 for sera positive for *P*. *papatasi*, *P*. *sergenti*, *P*. *tobbi*, *Cu*. *molestus* and for the negative control sera; and at 1:500 for sera positive for *P*. *perniciosus*. The strips were then incubated for 1 hour at room temperature with secondary antibodies (polyclonal anti-mouse or anti-dog IgG-HRP, AbD Serotec/ Bethyl laboratories)–diluted at 1:1000 in TBS-Tw–after which the chromogenic reaction was developed using 3,3′-diaminobenzidine in TBS with 0.03% H_2_O_2_. Distilled water was added to the strips to stop the reaction.

### Statistical analyses

In the SGH-ELISA all samples were run in duplicate and retested when a coefficient of variation (CoV) of more than 15% was obtained. Sera of positive (PC) and negative control (NC) dogs were included in each plate to correct for interplate variability. OD values were standardized (SOD) according to the following formula:

$$SOD\;(\%)=\frac{OD\;sample}{Average\;OD\;PC-average\;OD\;NC}x\;100$$

In all subsequent analyses the SGH-ELISA was set as the gold standard to which the results of the rSP03B sero-strip were evaluated. The seroprevalence of bitten dogs per region and per month was calculated from the SOD (%) SGH-ELISA results using a cut-off value for positivity according to the mean SOD (%) values from non-bitten negative control dogs plus two standard deviations (SD). Thereafter, expected positive and negative predictive values (PPV and NPV, respectively) were computed per region using the known sensitivity and specificity of the optimized rSP03B sero-strip. Consequently, all samples were also analyzed with the rSP03B sero-strip. The resulting signal was classified into four categories according to the intensity of the observed band, analogous to what has been proposed previously [20]. Samples classified in category (0) were considered negative, the ones classified in categories (2), (3), and (4) were considered positive. Since samples in category (1) merely showed a faint background signal, they were considered either positive or negative. The results of all samples when screened with the rSP03B sero-strip were converted into a proportion of dogs that were classified as being positive per region or per time point and graphically visualized using the “ggplot2” package in R software [24,25].

The degree of agreement between the SGH-ELISA and the rSP03B sero-strip was measured per region by Cohen’s Kappa according to the methods of Jacob Cohen [26]. The Mc Nemar’s χ^2^ test in R software [24] was applied to test for significant differences in agreement between these two methods. In order to assess the consistency in the ratings of signal intensity across samples between different readers or performers, the inter-person reliability was evaluated. Similarly, the reproducibility of the rSP03B sero-strip was assessed so to assure consistency in ratings over time. Both the inter-person reliability and reproducibility were computed using a two-way mixed, consistency, single-measures intraclass correlation coefficient (ICC) [27]. Moreover, the random effects single-measures model was used in order to allow generalization of the results rated by a single reader. Finally, the ICC was also computed to compare the performance of the ICT when run with serum versus whole canine blood.

## Results

### Predictive values of the optimized rSP03B sero-strip

The sensitivity and specificity of the optimized rSP03B sero-strip were determined based on the test results of dogs experimentally exposed to *P*. *perniciosus* female sand flies and samples coming from non-bitten laboratory-bred Beagles. In 3 out of 60 dogs experimentally exposed to *P*. *perniciosus* no band appeared on the test line when run with the rSP03B sero-strip, allocating a sensitivity of 95% (95% CI 85.18% - 98.70%) to this optimized ICT. Furthermore, in 3 out of 59 unexposed dogs a band appeared on the test line, giving it a specificity of 94.92% (95% CI 84.94% - 98.68%). Seroprevalence of bitten dogs per region were defined based on the SOD (%) obtained by SGH-ELISA and are given in Table 1. The cut-off for ELISA was calculated according to the mean SOD (%) values from non-bitten negative control dogs plus two standard deviations (SD) (equaling 34.74 SOD (%)). The positive and negative predictive values (PPV and NPV, respectively) of the rSP03B sero-strip were calculated per region based on the previously calculated seroprevalence of bitten dogs and the known sensitivity and specificity of the rSP03B sero-strip and are shown in Table 1.

**Table 1:** Seroprevalence of bitten dogs by ELISA and predictive values of the rSP03B sero-strip per region.

Regions	Seroprevalence of bitten dogs (%)	PPV (%)	NPV (%)
**Campania July 2012**	8.57	62.87	99.51
**Umbria August 2013**	14.29	75.06	99.13
**Umbria Oct 2013**	15.38	76.66	99.05
**Montagut July 2017**	33.33	90.03	97.43
**Montagut Sept 2017**	34.62	90.53	97.28
**Canet d'Adri July 2017**	75.00	98.19	86.33
**Canet d'Adri Sept 2017**	9.09	64.36	99.48
**Colonia de Sant Jordi Oct 2017**	41.67	92.81	96.37

The seroprevalence of bitten dogs per region was calculated based on the SOD (%) obtained by SGH-ELISA and was used together with the sensitivity and specificity of the rSP03B sero-strip to calculate the PPV and NPV per region. PPV, Positive Predictive Value; NPV, Negative Predictive Value.

### Inter-person reliability and reproducibility of the rSP03B sero-strip

The inter-person reliability was tested by running 30 randomly chosen sera samples with the rSP03B sero-strip either using two different raters or two different performers. The ICC was calculated for all combinations and is shown in Table 2. When the results of the two performers were compared, ICC values of 0.865 and 0.881 were obtained. Furthermore, comparing two different raters resulted in ICC values of 0.966 and 0.956. The reproducibility of the rSP03B sero-strip was ascertained by testing 30 sera samples on two different days. The ICC between both measurements was 0.93 (95% CI: 0.858–0.966; P-value: 2E-14).

**Table 2:** Intraclass Correlation Coefficients for inter-person reliability.

	Performer A vs. B				Rater A vs. B		
Constant	ICC	95 CI	P-value	Constant	ICC	95 CI	P-value
**Rater A**	0.865	0.737–0.934	1.68E-10	**Performer A**	0.966	0.929–0.983	8.34E-19
**Rater B**	0.881	0.766–0.942	3.07E-11	**Performer B**	0.956	0.909–0.979	2.92E-17

The ICC values obtained after comparing the results of the rSP03B sero-strip when two different performers were used are shown on the left; the ICC values obtained when the results of two different raters are compared are shown on the right. ICC, Intraclass Correlation Coefficient; CI, Confidence Interval.

### Optimization with whole canine blood

All 30 spiked blood samples from experimentally exposed dogs appeared positive on the test together with 4 out of 29 spiked blood samples from non-bitten laboratory dogs, giving the test with whole canine blood a sensitivity of 100% (95% CI 85.87% - 100%) and a specificity of 86.21% (95% CI 67.43%– 95.49%). The ICC between the results of sera collected in different regions endemic for CanL and whole canine blood spiked with the same sera samples was calculated and equals 0.866 (95% CI 0.822–0.9; P-value: 6.56E-51).

### Field detectability of the rSP03B sero-strip

**Fig 1:**
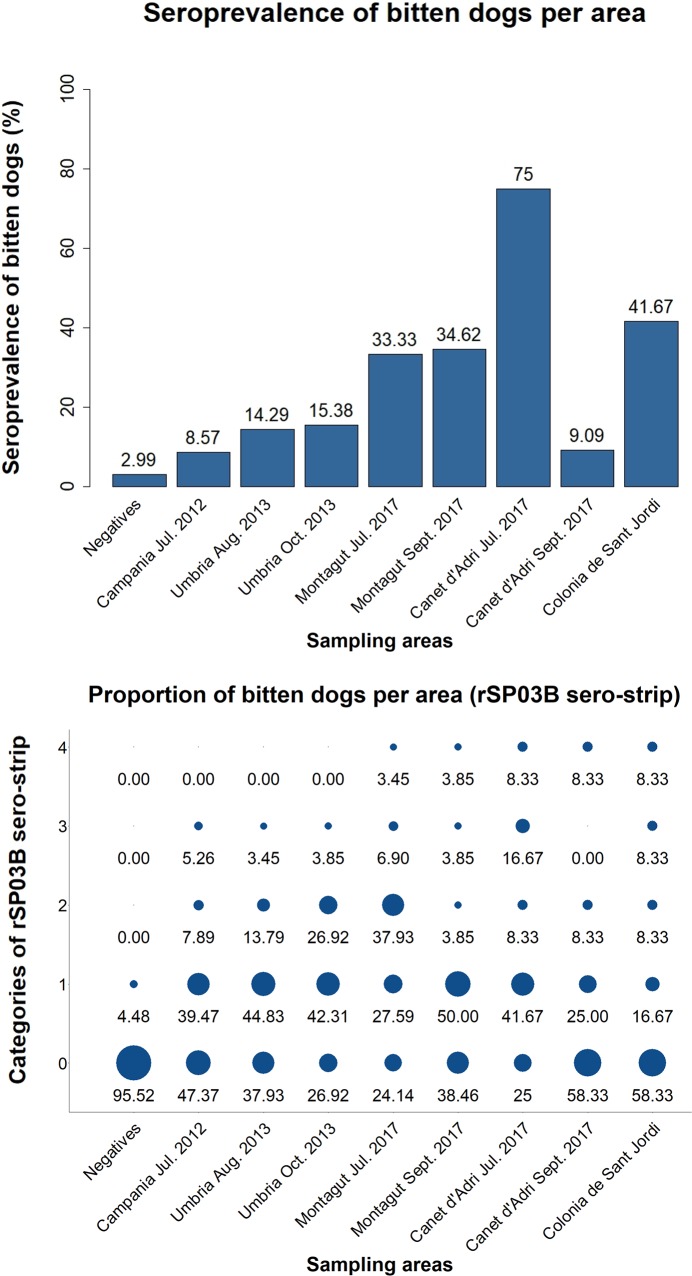
Graphical representation of results obtained per region. (A) Seroprevalence (%) calculated based on the SGH-ELISA results are plotted per region. (B) Results obtained by the rSP03B sero-strip are visualized per region and plotted according to the different categories of the test, starting from (0) a negative test result; (1) a very faint signal; (2) a low positive signal; (3) a positive but less intense signal than the control band and (4) a strong positive signal, as intense as the control band. The size of the plotted circle represents the proportion of dogs classified per category.

Both the seroprevalence calculated based on the SGH-ELISA results as well as the results from the rSP03B sero-strip were plotted per region and are shown in Fig 1. When assuming samples from category (1) to be positive, no significant systematic differences were observed between the proportions of positive responses obtained by these two methods (McNemar χ2 P > 0.05) for a total of four out of eight regions tested. The Cohen’s kappa value between the rSP03B sero-strip and the SGH-ELISA was calculated for each region and suggests an almost perfect strength of agreement between these two methods for one out of the eight regions tested. A fair degree of agreement was obtained for an additional two regions; for the other regions only a slight or no degree of agreement was obtained. However, when samples in category (1) were considered negative, the Cohen’s kappa value between the rSP03B sero-strip and the SGH-ELISA suggested a substantial degree of agreement between these two methods for one region, a fair degree of agreement for four out of eight regions, and only a slight or no degree of agreement for the other regions. Even more so, McNemar χ2 tested no systematic difference between the proportions of positive responses obtained by the SGH-ELISA or the rSP03B sero-strip for all regions tested. The outcome of the McNemar χ^2^ test and the Cohen’s kappa values for both analyses are shown in Table 3.

**Table 3:** Comparison of the rSP03B sero-strip with SGH-ELISA per region.

	**Category (1) is positive**		**Category (1) is negative**	
	**Kappa**	**McNemar** **P-value**	**Kappa**	**McNemar** **P-value**

**Campania July 2012**	0.196	0.000	0.160	**0.688**
**Umbria August 2013**	0.195	0.000	0.340	**1.000**
**Umbria October 2013**	0.126	0.000	0.371	**0.219**
**Montagut July 2017**	0.093	0.003	0.333	**0.289**
**Montagut Sept 2017**	0.210	**0.065**	0.194	**0.070**
**Canet d'Adri July 2017**	1.000	**1.000**	0.286	**0.063**
**Canet d'Adri Sept 2017**	0.214	**0.125**	0.621	**1.000**
**Colonia de Sant Jordi October 2017**	NA	**1.000**	NA	**0.688**

Degree of agreement between the rSP03B sero-strip and the SGH-ELISA is shown. Two analyses were performed based on classifying category (1) as positive or negative. A fair degree of agreement according to Cohen’s kappa is highlighted in light grey (0.21–0.40), a substantial degree of agreement is highlighted in darker grey (0.61–0.80), and an almost perfect degree of agreement is highlighted in dark grey (0.81–1.00). P-values for areas for which McNemar χ^2^ test did not detect a significant systematic difference between the outcome of the rSP03B sero-strip and the SGH-ELISA are highlighted in bold. NA, Not Applicable.

### Seasonal fluctuation of proportion of bitten dogs according to the rSP03B sero-strip

**Fig 2:**
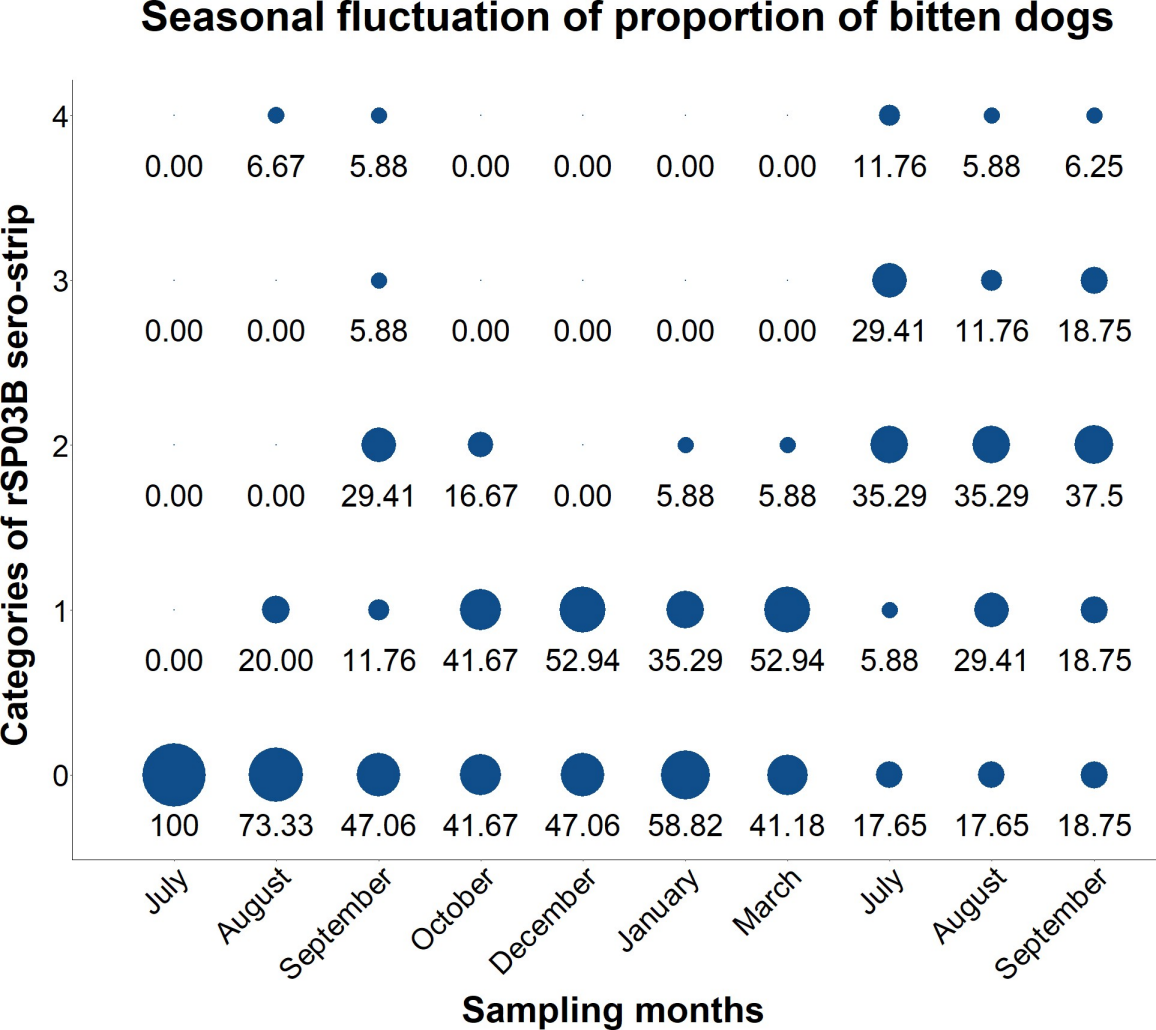
Graphical representation of results obtained with the rSP03B sero-strip during longitudinal sampling of dogs. Results obtained by the rSP03B sero-strip are visualized per month and plotted according to the different categories of the test to show the seasonal fluctuation of the proportion of bitten dogs. Category (0) represents a negative test result; (1) a very faint signal; (2) a low positive signal; (3) a positive but less intense signal than the control band and (4) a strong positive signal, as intense as the control band. The size of the plotted circle represents the proportion of dogs classified per category.

Results of the rSP03B sero-strip when naturally exposed dogs sampled multiple times during two transmission seasons were screened are visualized in Fig 2 and show a clear fluctuation, with a peak at months September–October of the first year of sampling and a second one during July–August of the second year. The highest proportion of positive dogs was observed during October (58.34%) of the first year and July (82.34%) and August (82.34%) of the second year. However, when considering samples in category (1) as negative, the highest proportion of positive dogs is observed for September (41.17%) of the first year and for July (76.46%), August (52.93%) and September (62.5%) of the second year. Even more so, this results in a clear drop in the total proportion of positive dogs from December to March, reaching zero percent in December and slowly increasing during January and March up to 5.88% for both months.

### Cross-reactions with other hematophagous arthropods

**Fig 3:**
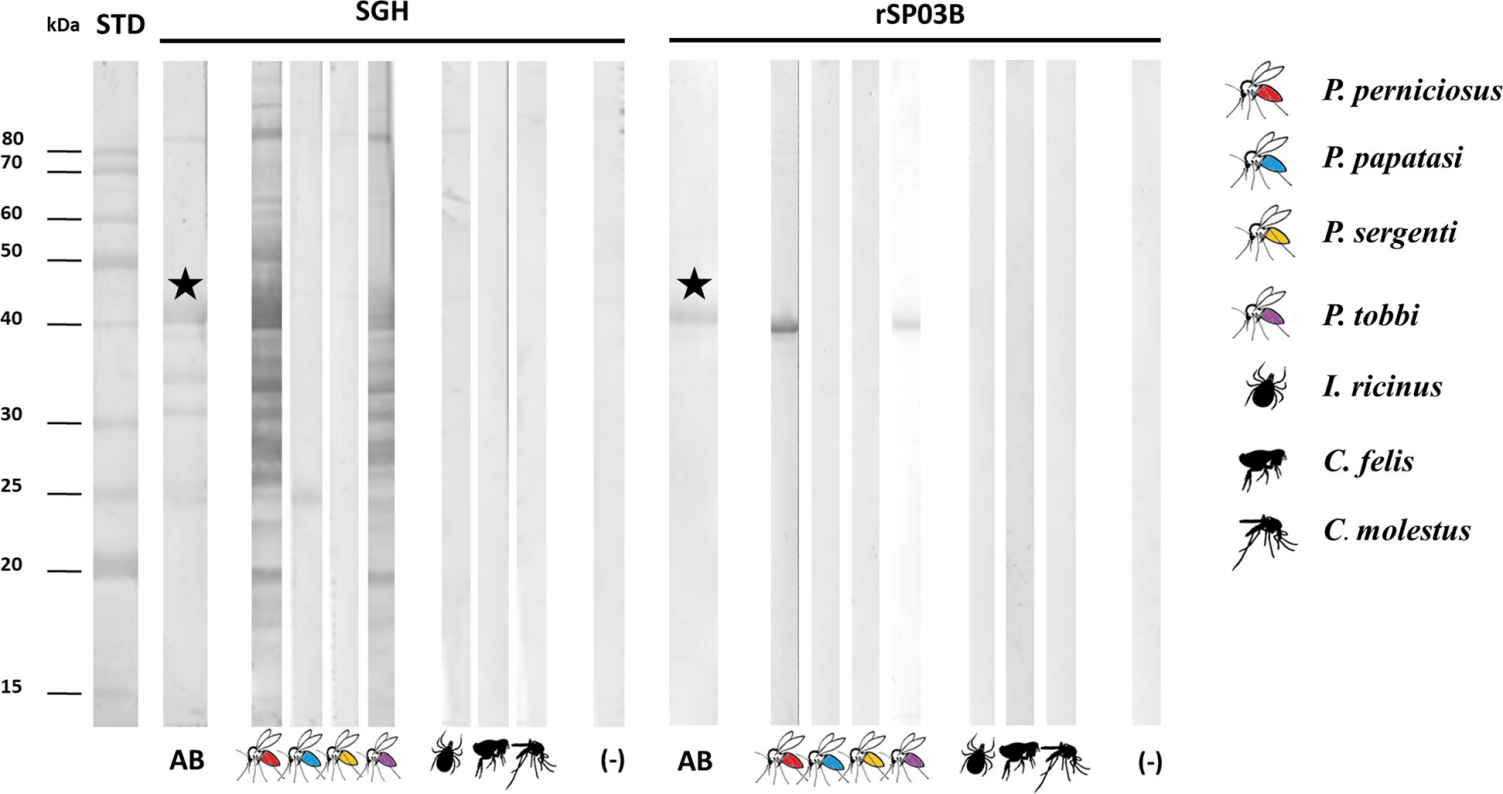
Western Blot. Results of a Western Blot performed with the SGH and the rSP03B protein of *P*. *perniciosus* and sera samples of hosts experimentally bitten by various hematophagous arthropods including *P*. *papatasi*, *P*. *sergenti*, *P*. *tobbi*, *C*. *felis*, *I*. *ricinus*, and *Cu*. *molestus*. Sera from non-bitten mice were used as negative control. The location of the rSP03B protein is indicated with a star sign. (-), negative control sera; STD, Standard; AB, Amidoblack stain.

Possible cross-reactions between anti-*P*. *papatasi*, anti-*P*. *sergenti*, anti-*P*. *tobbi*, anti-*Cu*. *molestus*, anti-*C*. *felis* and anti-*I*. *ricinus* IgG and the salivary proteins of *P*. *perniciosus* were assessed by performing a western blot with both the SGH and the rSP03B protein of *P*. *perniciosus*. The results are shown in Fig 3 and indicate that most sera samples do not interact with *P*. *perniciosus* salivary proteins located within the 15–80 kDa range. Cross-reactions were only observed between a protein of *P*. *perniciosus* of approximately 25 kDa and *P*. *papatasi* IgG Abs and between mice anti-*P*. *tobbi* IgG Abs and all tested salivary antigens from *P*. *perniciosus*.

## Discussion

We present a significant improvement of the rSP03B sero-strip previously demonstrated to be a valid replacement for the standard SGH-ELISA to measure levels of specific anti-vector salivary antibodies during epidemiological studies [20]. In order to ascertain proper functionality of the test in field conditions we have addressed additional specifications of our ICT. First, the ICT was optimized using the SP03B recombinant protein expressed in a human cell line (HEK 293) previously used for successful expression of yellow-related salivary proteins of *L*. *longipalpis* [16,17]. Recombinant proteins expressed with the HEK 293S GnTI- system lack complex N-glycans and hence resemble more precisely the insect’s N-glycosylation pattern. A previously optimized protocol for high-density transfection of the suspension adapted HEK293S GnTI^-^ cell line was followed [23]. The succeeding rSP03B sero-strip rendered an increased specificity (94.92%) compared to the previously proposed ICT while maintaining a high sensitivity value (95%). The increase in specificity of this optimized ICT is most likely due to the proper protein folding and post-translational modifications introduced by producing the protein in HEK293 cells.

Further optimization of this ICT required evaluating the inter-person reliability and reproducibility of the test by computing the ICC for both measurements, and optimizing it with whole canine blood. The obtained ICC values were interpreted according to the rules of Cicchetti et al [28]. Both the inter-rater reliability and the reproducibility ICC values were in the excellent range indicating that different individuals rating or performing the ICT as well as ratings obtained on different days have a high degree of agreement. These results signify that only a minimal amount of measurement error is introduced. Consequently, signal intensity ratings were considered to be suitable for use in the present study. Final optimization of the ICT for the use of whole canine blood required minor adjustments and resulted in an excellent degree of agreement between the use of sera and whole canine blood.

In a third part of this study, we have made a first attempt to address the field detection accuracy of the optimized test by (a) screening serum samples from canine populations exposed to various frequencies of sand fly bites with both the optimized rSP03B sero-strip and the SGH-ELISA and (b) testing possible cross-reactions with other hematophagous arthropods present across the Mediterranean area using sera of experimentally bitten hosts.

Since in our previous study we confirmed that the SGH-ELISA is the most reliable method to evaluate the extent to which dogs have been bitten by *P*. *perniciosus*–with sensitivity and specificity values equaling 100% and 98.11%, respectively [20]–we continued to use this method as the gold standard against which the rSP03B sero-strip is evaluated. Therefore, the results obtained by the SGH-ELISA were used to estimate the seroprevalence of bitten dogs per screened area. These results indicate that only four areas have a seroprevalence of bitten dogs high enough (> 20%) in order to allow an optimal representation of the predictive values of the rSP03B sero-strip in the field, as is indicated by higher PPVs for these areas. Results obtained by the rSP03B sero-strip were translated into a proportion of bitten dogs and compared to the seroprevalence per region. The results indicate that samples classified in category (1) represent a grey zone, comprising both true positive dogs (in areas with a high seroprevalence) as well as negative dogs (in areas with a low seroprevalence). When excluding these samples from the analysis, all areas showed an agreement between the ICT and the SGH-ELISA (McNemar χ2) and for six out of eight areas a certain degree of agreement was observed (Cohen’s kappa). Interestingly, the degree of agreement according to Cohen’s kappa increases for areas with a low seroprevalence of bitten dogs, whereas it decreases for areas with a high seroprevalence of bitten dogs. This can be explained by the high proportion of dogs classified in category (1). Furthermore, longitudinally collected canine sera samples showed a clear seasonal fluctuation for the proportion of bitten dogs. A previous study by Kostalova et al [14] investigated the antibody responses in these dogs with the SGH-ELISA and found significant differences between months from July until October in the first year of sampling and from March until July in the second year of sampling. This increase in antibody response was suggested to reflect an increased biting rate during these months and is in line with the peak of sand fly abundance in the area. In the present study, we were able to relate these peaks in antibody response to both higher signal intensities observed with the ICT and consequently to a higher proportion of dogs classified as being positive. A more pronounced fluctuation in the proportion of bitten dogs was observed when the grey zone–as explained before–was excluded from the analysis. Taken together, these results indicate that the high sensitivity and specificity of the ICT enable it to visualize the seasonal fluctuation of the antibody response against *P*. *perniciosus* salivary proteins and hence support its use in the field, particularly in areas with a high seroprevalence of bitten dogs–bearing in mind however, that for samples which show a weak background signal on the ICT re-evaluation of the results by another method or the use of an automated strip reader is recommended (reviewed in [29]).

A second step in confirming the field detection accuracy of the optimized test was to evaluate possible cross-reactions between the anti-saliva antibodies of other bloodsucking arthropods and salivary proteins of *P*. *perniciosus*. It is believed that salivary proteins from phylogenetically closely related sand fly species will likely cross-react with each other, as has been described in previous studies [30,31]. Vectors of canine leishmaniasis co-occurring with *P*. *perniciosus* in the western part of Mediterranean Europe comprise *P*. *ariasi*, *P*. *perfiliewi* and *P*. *neglectus* [2], all being members of the *Larroussius* subgenus. Due to a lack of colonies of most of these sand fly species [32] testing their cross-reactivity is challenging. Therefore, in order to get an idea on these favorable cross-reactions *P*. *(Larroussius) tobbi* was included in our study; a vector of *L*. *infantum* in the eastern part of the Mediterranean basin, of which the distribution does not overlap with *P*. *perniciosus* [33,34]. The two yellow-related proteins of *P*. *tobbi* (Genbank accn. HM140619.1 and HM140618.1) are 90.59% and 70.44% identical to the 43 kDa yellow-related protein of *P*. *perniciosus* (Genbank accn. DQ150622), suggesting that cross-reactions between them are plausible. Indeed, this study confirms the presence of cross-reactions between salivary proteins across members of the subgenus *Larroussius*, vectors of canine leishmaniasis. On the contrary, we confirmed the absence of unspecific interactions between *P*. *perniciosus* and other hematophagous arthropods, including two sand fly species–*P*. *papatasi* and *P*. *sergenti*–which are refractory to *L*. *infantum* [35,36], thereby affirming the use of salivary antigens from *P*. *perniciosus* to detect canine vector exposure without any background signal caused by unspecific interactions with non-vector species.

In summary, we have successfully optimized the previously proposed rSP03B sero-strip by using the rSP03B protein expressed in a human cell line which delivered a higher specificity of the test. Additionally, signal intensity ratings were equal across performers as well as raters and the reproducibility of the ICT was excellent, proving that future use of the ICT will not be influenced by these factors. The ICT has been optimized for use with whole canine blood and results from the field assessment confirm its functionality in the field and even proved the presence of favorable cross-reactions with other sand fly vectors of canine leishmaniasis. Hence, this improved rSP03B sero-strip represents a valuable tool in monitoring dog exposure to vectors of CanL and may contribute significantly to future control programmes.
